# Spiral tracing on a touchscreen is influenced by age, hand, implement, and friction

**DOI:** 10.1371/journal.pone.0191309

**Published:** 2018-02-01

**Authors:** Brittany D. Heintz, Kevin G. Keenan

**Affiliations:** 1 Department of Kinesiology, University of Wisconsin–Milwaukee, Milwaukee, WI, United States of America; 2 Center for Aging and Translational Research, University of Wisconsin–Milwaukee, Milwaukee, WI, United States of America; University of Zurich, SWITZERLAND

## Abstract

Dexterity impairments are well documented in older adults, though it is unclear how these influence touchscreen manipulation. This study examined age-related differences while tracing on high- and low-friction touchscreens using the finger or stylus. 26 young and 24 older adults completed an Archimedes spiral tracing task on a touchscreen mounted on a force sensor. Root mean square error was calculated to quantify performance. Root mean square error increased by 29.9% for older vs. young adults using the fingertip, but was similar to young adults when using the stylus. Although other variables (e.g., touchscreen usage, sensation, and reaction time) differed between age groups, these variables were not related to increased error in older adults while using their fingertip. Root mean square error also increased on the low-friction surface for all subjects. These findings suggest that utilizing a stylus and increasing surface friction may improve touchscreen use in older adults.

## Introduction

Most portable electronic devices require manipulation of touchscreens (e.g., laptops, mobile phones, and tablets), which involves coordination of downward pressing forces and movements. Motor control studies typically assess force and motion tasks separately [1–3], with age-associated changes on either task well documented in older adults [1, 2, 4–6]. Moreover, the coordination of force and motion during hybrid tasks presents a relatively greater motor control challenge, as force and motion tasks are thought to have independent neural origins [7, 8]. Thus, older adults find manipulation of technological devices challenging and age-associated impairments contribute to decreased use of technology, limiting associated benefits in this population including decreased life-stress, increased social support, well-being, self-efficacy, sense of empowerment, and functional independence [9–16].

In addition to age-associated differences in manipulating touchscreens, multiple other factors could influence touchscreen manipulation. First, low-friction surfaces, such as those found on many touchscreens, lead to impaired motor performance [2, 17]. For example, performing tasks against low- vs. high-friction surfaces leads to decreased performance on the Box and Block test [17], changes in precision gripping [18, 19], and increased force fluctuations [2]. Furthermore, the coefficient of friction between the finger and touchscreen can vary over a wide range by applying different screen protectors, which can also influence performance [20]. Second, motor performance is dependent on hand used [21, 22], and left-hand use by right-handed individuals sometimes leads to decreased dexterity [21, 23], though this is not always the case in older individuals [24]. Third, drawing implement (i.e. index fingertip vs. stylus) impacts performance on touchscreens. Performance has been shown to improve at times for young [25] and older adults [26] using a pen vs. index finger, with performance generally better using a stylus in the dominant hand [27]. Conversely, some studies show performance improvements with the index finger vs. stylus or varying performance depending on task constraints [28, 29]. How these multiple factors (i.e., friction, hand use, and drawing implement) interact with age to influence touchscreen manipulation is not clear.

Thus, the purpose of our study was to examine differences in young and older adults tracing an Archimedes spiral on a touchscreen while swiping against high- and low-friction surfaces using the finger or a stylus as the drawing implement. In the current study, our aim is to assess age-associated changes in motor performance on a clinical Archimedes spiral tracing task implemented on a touchscreen to quantify position accuracy during the hybrid task. This test has been shown to be a sensitive measure of differences between young and older adults [30, 31], is frequently used clinically to evaluate tremor [32–35], and can be performed using a digitizing tablet [30, 32, 35, 36]. Our hypothesis, based on the previously cited literature, is that older vs. young adults will exhibit decreased performance on the Archimedes spiral tracing task, especially while performing the task with their left vs. right hand, with the index finger vs. stylus, and while interacting on a low- vs. high-friction surface.

Additional factors known to change with age were assessed to examine potential mechanisms associated with impairments in touchscreen manipulation. To identify if young and older adults used different motor strategies, we quantified the downward force that each subject pressed with and completion time. Older adults have demonstrated increased forces during precision gripping and impairments in force control [2, 18, 19], therefore we expect that older vs. young adults will press with greater downward force. In addition, we assessed fingertip sensation with the von Frey index and visuomotor function with simple reaction time (SRT) and choice reaction time (CRT), and their associations with touchscreen manipulation. Age-related impairments are well established for SRT and CRT [37, 38], while sensation decreases with age and is suggested to be related to fine motor control [39]. Declines in visuomotor processing and fingertip sensation may be important for touchscreen manipulation, thus, we hypothesize that age-related impairments on these measures will be related to spiral tracing performance.

## Materials and methods

### Ethics statement

The experiments were approved by the Institutional Review Board at the University of Wisconsin–Milwaukee. The local ethics committee approved consent for subjects ranging from 18–40 years and 65–89 years. Written informed consent was obtained from all individual participants included in the study.

### Participants

A total of 50 subjects, 26 young (age: 24.0 ± 3.8 years; range, 19–34 years; 15 females) and 24 older adults living independently (age: 73.9 ± 6.4 years; range, 65–89 years; 14 females), volunteered. All participants were right-handed as assessed by the Edinburgh Handedness Inventory [40], and had normal or corrected-to-normal vision, with no reported neuromuscular disorders, carpal tunnel syndrome, previous upper extremity surgeries, diabetes, peripheral neuropathies, upper extremity pathologies impacting hand use or dexterity, or use of current medications known to influence neuromuscular control.

### Experimental arrangement and procedures

#### Spiral tracing

**Fig 1:**
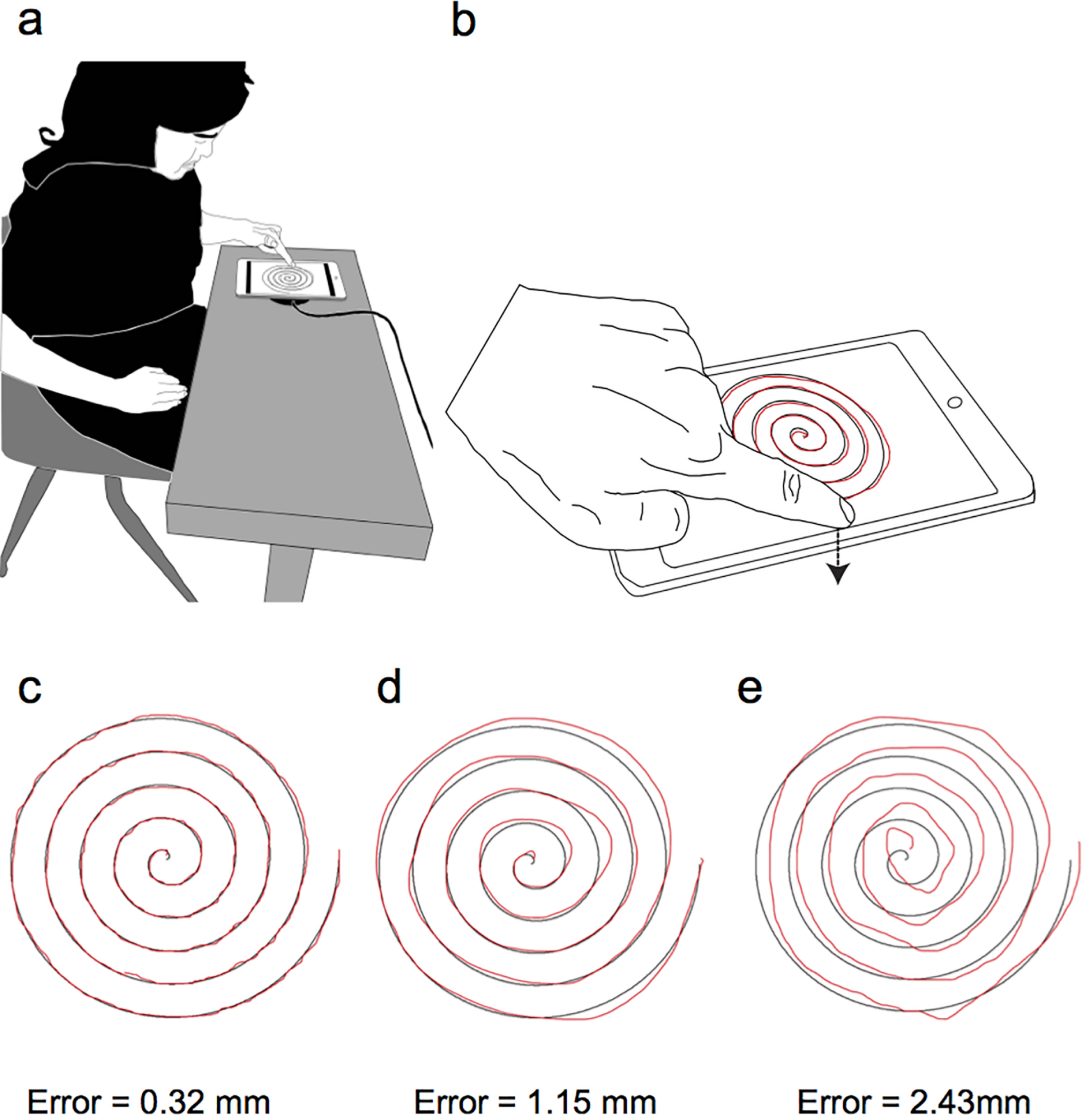
Experimental setup for the spiral tracing task and representative data. (*a*) The subject’s seated position relative to the touchscreen tablet, and (*b*) the spiral template (black) and subject’s spiral trace (red) as it appeared on the tablet touchscreen for a left hand trial. Subjects pressed onto the touchscreen tablet, which was mounted on a force sensor measuring downward forces. Representative data from three right handed trials with varying performance. Subject traces from three participants (red) are superimposed on the spiral template (black) resulting in (*c*) low, *(d*) average, and (*e*) high RMS error.

Subjects were seated in front of an adjustable metal table with a horizontal platform (69 cm high). Forces were recorded at 50 samples/s with a six-axis force/torque sensor (Gamma, ATI Industrial Automation, Apex, NC) rigidly fixed in the center of the table and connected to a portable laptop (Dell Latitude E5430, Round Rock, TX). The sensor plane was oriented horizontally, with the normal force directed downward. An iPad mini (13.4 cm x 20.0 cm x .72 cm, Apple Inc., Cupertino, CA,) with an LED-backlit Multi-Touch display (1024 x 768 resolution, 163 pixels per inch, 20.07 cm diagonal) was centered and rigidly mounted on the force sensor (Fig 1A).

Subjects traced over an Archimedes spiral displayed as a black line (0.42 mm thickness) on the iPad touchscreen (Fig 1B). Contact with the touchscreen and subsequent line tracing was completed using: 1) the index finger or a stylus (Bamboo Solo CS100K, Wacom, Portland, OR), 2) left or right hand, and 3) a low- or high-friction contact surface. The low-friction surface was created by applying a screen protector (Spigen SGP, Spigen, Inc., Irvine, CA) to the touchscreen surface, while the high-friction surface was the original iPad glass. The dynamic coefficient of friction (CoF) was calculated previously in our lab [20] to establish the low- and high-friction conditions (CoF: Spigen SGP = 0.54 ± 0.21; iPad glass = 1.48 ± 0.46). Feedback while tracing was provided via a red line (0.42 mm thickness) superimposed onto the black spiral image on the iPad (Fig 1B). Subjects were asked about their comfort level and ability to see the image projected on the iPad mini. No one expressed concerns or asked us to change table height to perform the task, and all subjects reported being able to clearly see both red and black lines. In piloting for this project, we placed the adjustable table at a height of 69 cm (i.e., 27 in), because the dimensions of the force sensor and iPad mini raised the drawing surface to a height of 28 in, which is within the standard height for most tables (i.e., 28–30 in).

The following instructions were provided to each subject. Begin in the center of the spiral working outwards and stay as close to the black line as possible, complete the spiral at the self-selected pace necessary to remain as close to the spiral line as possible, and avoid lifting the finger or stylus once the trial begins. Touchscreen contact was made with the stylus or index finger, and the hand and wrist were not allowed to rest on the touchscreen surface. The subject was free to move their upper extremity, as long as the drawing implement did not leave the touchscreen and a second point of contact was not made. The skin of the subject’s index fingertips was cleaned with ethanol before each experiment to ensure a clean contact point. In addition, the touchscreen surface was cleaned between all conditions with a microfiber cloth. Practice trials were administered prior to each new condition to ensure that participants were comfortable with the task. Three trials for each experimental condition were completed. The sequence of trials was block randomized across experimental conditions.

#### Reaction time

Participants were seated facing a portable laptop computer (Dell Latitude E5430, Round Rock, TX) placed on a table with the keyboard and display (1366 x 768 HD resolution, 14.0 inch diagonal, Anti-Glare LED) at a comfortable distance in front of the participant. Two reaction time tests were completed to assess visuomotor function, SRT and CRT. Both tests are commonly included in studies assessing visuomotor processing to examine both relatively simple and complex cognitive processes [37, 38]. The tests were created using custom scripts in the Psychophysics Toolbox for Matlab (The MathWorks, Inc., Natick, MA).

For SRT, participants placed their right index finger on the ‘Space’ bar and viewed a blank, white display. Instructions were to respond to the cue, which appeared as a black rectangle in the center of the screen, as quickly as possible by pushing the ‘Space’ bar with the index finger. The cue appeared at various intervals (every 200–400 ms) after the termination of the previous trial. SRT was calculated as the time from the appearance of the stimulus to the time the participant responded by pressing the ‘Space’ bar. 10 practice trials were completed prior to 20 SRT trials. The average time across all 20 trials was calculated.

For CRT, participants placed their right and left index fingers on the right and left ‘Shift’ keys, respectively. A blank, white screen was presented on the laptop display, with the words ‘RED’ and ‘BLUE’ in the bottom left or right corner of the display. The position of each word (i.e. bottom left or bottom right) was randomized between trials. The rectangular cue appeared at various intervals (every 200–400 ms) as either a red or blue rectangle in the center of the screen. Instructions were to push the ‘Shift’ key on the right or left side that corresponded with the correct color of the rectangle as quickly as possible. 10 practice trials were performed prior to the test trials. 20 CRT test trials, 10 red and 10 blue, were performed with the colors presented in a randomized order. A short break was provided after trial 10 to avoid fatigue. The average time across all 20 trials was calculated.

#### Von Frey index

Index fingertip sensation was determined using the standardized von Frey Anesthesiometer (Lafayette Instrument Co., Lafayette, IN) [41] to determine whether age-associated impairments in sensation were related to touchscreen manipulation. Subjects were seated with the upper extremity resting on a Versaform Pillow (Tumble Forms, Dolgeville, NY) on a table in the supinated position with the index finger accessible. The von Frey filament was pressed at a 90-degree angle against the palmar surface of the distal phalanx of the index finger for approximately 1.5 seconds then removed. Subjects signaled when they felt the stimulus by saying ‘touch’, with eyes closed during testing. Three increasing and decreasing positive stimulus identification values were recorded, alternating between the two types until all trials were complete. An increasing value was the first filament felt when increasing from smallest to largest filament size, and a decreasing value was the last filament felt while decreasing from largest to smallest filament size. Trials were randomized between hands. The mean of the six trials was calculated for each hand to find the von Frey index. Higher von Frey index corresponds to decreased sensation. The order of all experimental tasks was randomized across participants.

#### Touchscreen usage

A brief survey was given to assess previous touchscreen usage. The participants were asked whether they had previously used or owned a device with a touchscreen prior to the testing session.

### Data analysis

Analysis for the spiral tracing tasks was performed using custom scripts in Matlab (The MathWorks, Inc., Natick, MA). To determine accuracy on the spiral tracing task, Root mean square (RMS) error was calculated to assess the proximity of the subject’s spiral trace to the original template, similar to the approach used by Marmon et al. (2011) and Hoogendam et al. (2014). The subject’s spiral trace was exported from the iPad as a PDF file using PDF-notes (AMuseTec Co., Ltd, Seoul, South Korea), then imported into Matlab for analysis. Each trace was compared to the original spiral template to calculate RMS error. Specifically, the line representing the original spiral template was composed of 916 pixels, and the radial distance between each pixel on the spiral template and the corresponding trace produced by the subject was calculated. The error for one trial was calculated as the average radial distance derived from the 916 values (Fig 1C–1E), reported in millimeters. The RMS error for each condition was the average for the three trials for each condition.

Downward force was measured using the force sensor positioned underneath the iPad. To calculate completion time for the spiral tracing task, the beginning and end of each trial was calculated from the time the downward force increased above 0.2 N to the time it decreased below 0.2 N, respectively. Mean downward force was calculated as the average force from the beginning to the end of each trial. Completion time and mean downward force were computed and averaged across the three trials for each condition.

### Statistical analysis

Data for RMS error, SRT, and CRT were moderately and positively skewed, therefore square root transformations were performed on this data prior to analysis [42] (SPSS 19.0, Chicago, Il). Normality of RMS error, SRT, and CRT were confirmed after transformations using both the Shapiro Wilk’s test and visual inspection of the Q-Q plots. For the spiral tracing task, one repeated measures ANOVA was performed with four factors. Dependent variables were RMS error, downward pressing force, and task completion time, and using within-subjects factors of friction condition, drawing implement, and hand used, and a between-subjects factor of age. Significant interactions were followed by post-hoc t-tests with Bonferroni corrections.

Pearson correlations were used to determine the relationship between RMS error using the fingertip and RMS error using the stylus for both age groups, with RMS errors collapsed across other conditions (i.e., hand and friction). The relationships between RMS error and other behavioral metrics were also assessed to determine whether these variables were related to age-associated changes in spiral tracing performance while using the fingertip, the condition that presented the greatest RMS error in the older adults. Specifically, Pearson correlations were used to determine the association between RMS error using the fingertip and von Frey index, SRT, CRT, completion time, and downward pressing force for both young and older age groups.

Two-sample, independent t-tests were used to examine differences between young and older adults for the von Frey index, SRT, and CRT. As anthropometrics could also influence results, a two-sample, independent t-test was used to assess if there were differences in height and weight of young and older adults. A two-sample, independent t-test was also used to assess differences in RMS error between older adults who previously used a device with a touchscreen and those who had not and between those who had previously owned a device with a touchscreen and those who had not. Young adults were not included in this analysis because all subjects in this age group reported previously using and owning a device with a touchscreen. Data is reported as the mean ± standard deviation in text and mean ± standard error in figures. For transformed data (i.e. RMS error, SRT, CRT), de-transformed means and 95% confidence intervals are reported. Alpha level for all statistical tests was *p* < 0.05.

## Results

### Participants

Average height was 172.00 ± 9.45 cm (range, 152.40–193.04 cm) and 169.97 ± 0.55 cm (range, 152.40–185.42 cm) for young and older adults, respectively. Average weight was 71.95 ± 16.36 kg (range, 48.99–127.01 kg) and 81.33 ± 18.19 kg (range, 49.90–117.48 kg) for young and older adults, respectively. There were no significant differences in height (*p* = .307) and weight (*p* = .061) between the two age groups.

### RMS error

**Fig 2:**
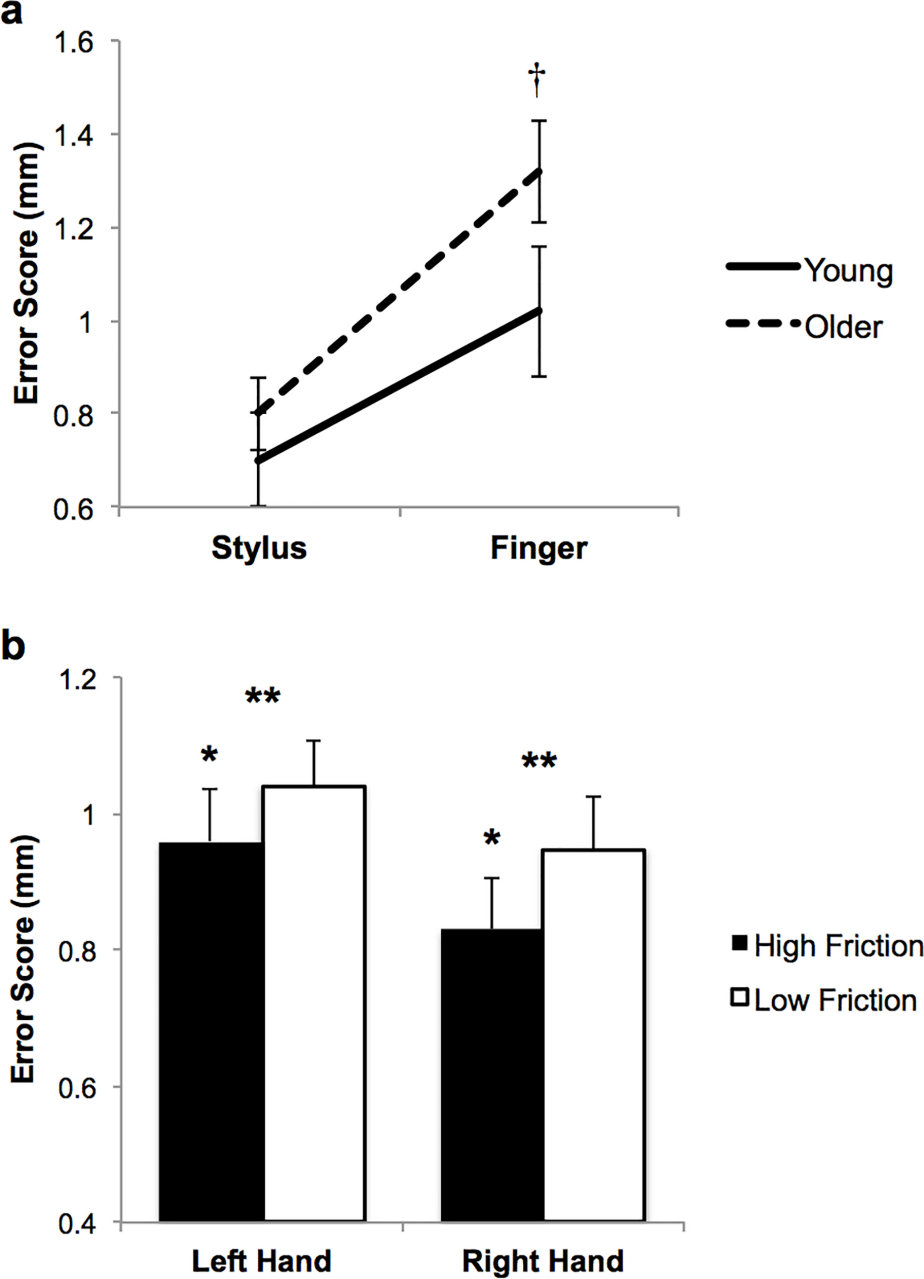
RMS error is influenced by age, drawing implement, touchscreen surface friction, and hand used. *(a)* There was a significant interaction between age and drawing implement for RMS error during the spiral tracing task (*p* = .010). While RMS error was greater (†*p* = .001) for the older vs. young adults when tracing with the index fingertip, RMS error for older adults decreased to levels similar to young adults when tracing with the stylus (*p* = .147). (*b*) RMS error increased for both young and older adults when tracing on the low- vs. high-friction touchscreen surface (**p < .001), and when tracing with the left vs. right hand (*p < .001). Data are shown collapsed across all other conditions.

There was a significant interaction for RMS error between age group and drawing implement (*F*(1, 48) = 7.149; *p* = .010, *η*_*p*_^*2*^ = .130; Fig 2A). Specifically, RMS error was greater for older vs. young adults while using the fingertip (*p* = .001, *η*_*p*_^*2*^ = .195; mean = 1.32 mm; 95% confidence interval [CI] = [1.18, 1.46] and mean = 1.02 mm; 95% CI = [0.91, 1.13], respectively), but with similar RMS error between young and older adults while using the stylus (*p* = .147; mean = 0.70 mm; 95% CI = [0.62, 0.79] and mean = 0.80 mm; 95% CI = [0.70, 0.90], respectively; Fig 2A). There was a significant main effect for RMS error for friction condition (*F*(1, 48) = 33.786; *p* < .001, *η*_*p*_^*2*^ = .413), with greater RMS error on the low- vs. high-friction touchscreen surface (mean = 0.99 mm; 95% CI = [0.92, 1.07] and mean = 0.89 mm, 95% CI = [0.83, 0.97], respectively; Fig 2B). A significant main effect for RMS error was found for the hand used (*F*(1, 48) = 30.952; *p* < .001, *η*_*p*_^*2*^ = .392), with greater RMS error using the left vs. right hand (mean = 1.00 mm; 95% CI = [0.93, 1.08] and mean = 0.89 mm; 95% CI = [0.82, 0.96], respectively; Fig 2B). All other main effects and interactions were not significant (*p* > .066).

**Fig 3:**
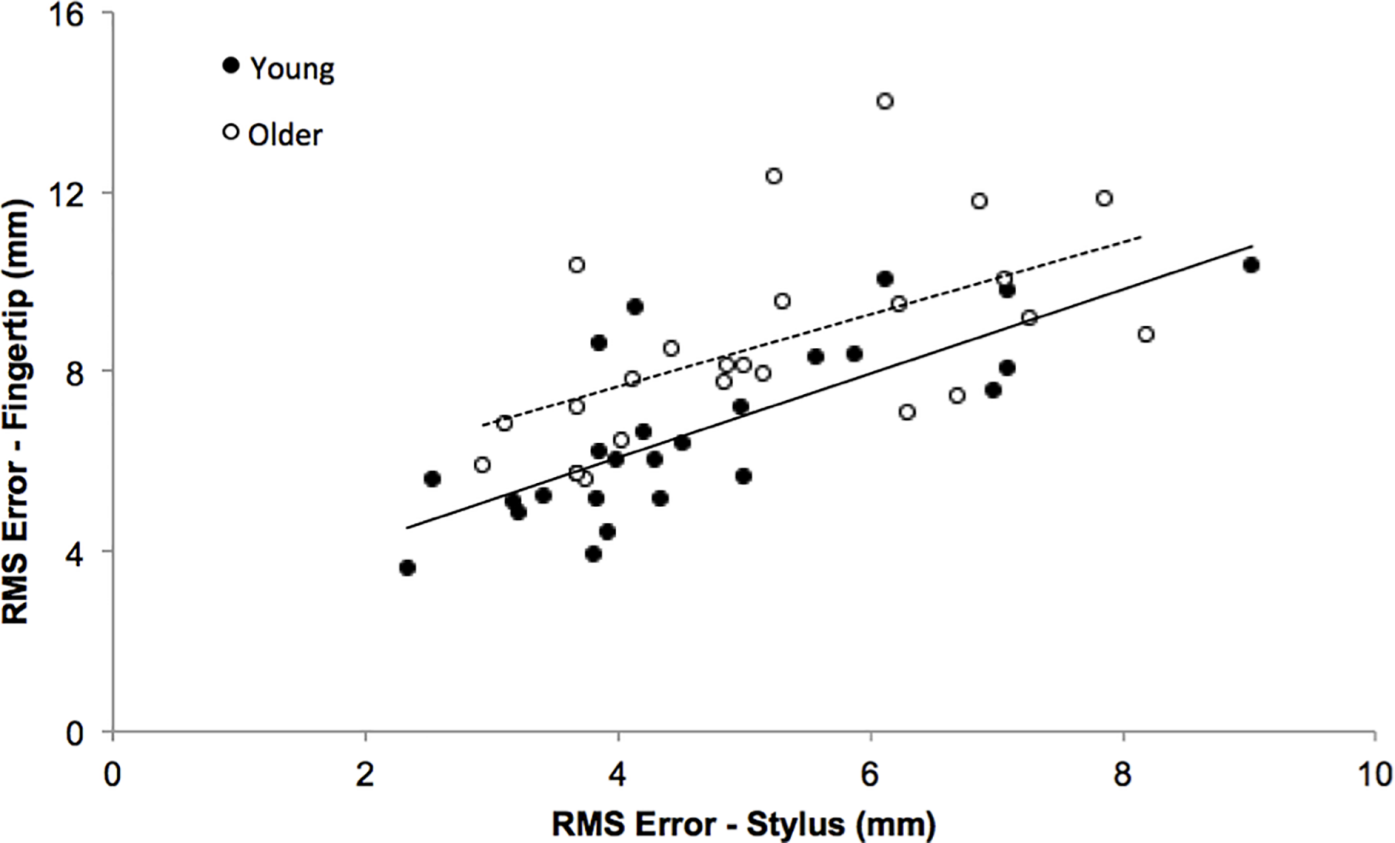
Relationship between RMS error on the spiral tracing task using the index finger vs. stylus. Though RMS error with the index fingertip and stylus were significantly correlated for the young (*r*^2^ = 0.588; *p* < .001) and older adults (*r*^2^ = 0.350; *p* = .002), the decreased correlation for older adults indicates that additional factors may be responsible for the age-related differences in RMS error while using the fingertip.

Further analysis was performed to explore potential factors that could explain why older adults performed worse during the spiral tracing task using their fingertip vs. stylus. RMS error using the fingertip was significantly correlated with RMS error using the stylus for the young adults (*r*^2^ = 0.588; *p* < .001), and although the correlation was significant for older adults (*r*^2^ = 0.350; *p* = .002), it was less than for young adults (Fig 3). As older adults performed the task as well as young adults with the stylus, the decreased correlation for older adults likely indicates that additional factors are responsible for the age-related differences in RMS error while using their fingertip. Consequently, numerous additional variables were analyzed to determine if they differed between young and older adults and to identify if specific factors were related to RMS error.

### Completion time

Time to complete the spiral tracing task was not different (*F*(1,48) = .806; *p* = .374) between young (35.32 ± 17.77 s) and older (30.80 ± 17.77 s) adults. Completion time was significantly greater (*F*(1,48) = 34.812; *p* < .001, *η*_*p*_^*2*^ = .420) when using the stylus (36.10 ± 18.21 s) vs. the index finger (30.02 ± 18.10 s) and was greater (*F*(1,48) = 6.934; *p* = .011, *η*_*p*_^*2*^ = .126) when using the left (34.05 ± 18.72 s) vs. right hand (32.07 ± 17.21 s). There were no other significant interactions that included age (*p* > .117).

### Downward pressing force

A significant main effect for downward pressing force during the spiral tracing task was found for age (*F*(1, 48) = 8.013; *p* = .007, *η*_*p*_^*2*^ = .143) with older adults pressing with more force than young adults (2.32 ± 1.13 N and 1.45 ± 1.04 N, respectively). A significant main effect for downward force was found for right vs. left hand (*F*(1, 48) = 47.940; *p* < .001, *η*_*p*_^*2*^ = .500), with greater pressing force using the left vs. right hand (2.12 ± 1.24 N and 1.65 ± 0.97 N, respectively). Downward force was also greater (*F*(1, 48) = 16.866; *p* < .001, *η*_*p*_^*2*^ = .260) while using the stylus vs. index finger (2.12 ± 1.08 N and 1.65 ± 1.22 N, respectively). There were no other significant effects or interactions (*p* > .082).

### Von Frey index and reaction times

Von Frey index was significantly greater (*t*(48) = 2.011; *p* = .001) for the older (3.75 ± 1.79) vs. young (2.33 ± 0.90) adults. Older adults had significantly greater SRT (*t*(48) = 2.010; *p* < .001) compared to young adults (mean = 0.34 s; 95% CI = [0.30, 0.39] and mean = 0.25 s; 95% CI = [0.24, 0.26], respectively). Additionally, older adults had significantly greater CRT (*t*(48) = 2.011; *p* < .001) compared to young adults (mean = 1.01 s; 95% CI = [0.89, 1.10] and mean = 0.68 s; 95% CI = [0.63, 0.72], respectively).

### Associations with spiral tracing performance

The relationships between spiral tracing RMS error and the five factors above (i.e., completion time, downward force, sensation, SRT, CRT) were computed to identify associations between increased error on the spiral tracing task for older adults while using the fingertip. Interestingly, no measure was related to RMS error for older adults (Table 1), though increased CRT and completion time were related to increased RMS error for young adults.

**Table 1:** Relationship between touchscreen performance using the fingertip and completion time, downward force, von Frey index, simple reaction time, and complex reaction time for young and older adults.

	Completion Time	Downward Force	Von Frey Index	Simple Reaction Time	Choice Reaction Time
**Young Adults**	*r*^2^ = 0.361	*r*^2^ = 0.010	*r*^2^ = 0.042	*r*^2^ = 0.000	*r*^2^ = 0.170
	*p* = 0.001	*p* = 0.636	*p* = 0.316	*p* = 0.992	*p* = 0.037
**Older Adults**	*r*^2^ = 0.096	*r*^2^ = 0.005	*r*^2^ = 0.031	*r*^2^ = 0.036	*r*^2^ = 0.060
	*p* = 0.141	*p* = 0.738	*p* = 0.408	*p* = 0.373	*p* = 0.249

### Touchscreen use

All young adults (n = 24) reported previously using and owning a device with a touchscreen. However, 11 older adults reported previously using a device with a touchscreen while 13 did not, and 7 older adults reported previously owning a device with a touchscreen while 17 did not. Interestingly, there was no difference (*t*(22) = 2.074; *p* = .329) in RMS error between older adults who had previously used and not used a touchscreen (mean = 1.02 mm; 95% CI = [0.86, 1.19] and mean = 1.12 mm; 95% CI = [0.97, 1.28], respectively). Furthermore, there was no difference (*t*(22) = 2.074; *p* = .230) in RMS error between older adults who had and had not owned a touchscreen (mean = 0.98 mm; 95% CI = [0.74, 1.24] and mean = 1.11 mm; 95% CI = [0.99, 1.24], respectively).

## Discussion

The main finding of the study was that both young and older adults had increased RMS error on the spiral tracing task while tracing with the fingertip compared to the stylus, though older adults had increased RMS error compared to young adults only while using the fingertip but not the stylus (Fig 2A). In addition, RMS error increased for both age groups while tracing on the low-friction surface and using the left hand (Fig 2B). Our novel implementation of the Archimedes spiral tracing task on a touchscreen tablet allowed us to assess performance while using the finger and stylus as drawing implements on a popular technological device and to manipulate the frictional properties of the interface. The Archimedes spiral tracing task has been previously reported to be a sensitive measure of age-associated changes in manual dexterity [30, 31], is used clinically to evaluate tremor [32–35], and is often completed with pen/pencil on paper [31, 33], or on a digitizing tablet [30, 32, 35, 36]. Our work suggests the need to carefully consider how the Archimedes spiral task is implemented to optimize detection of age-associated changes.

The Archimedes spiral is normally traced with a pen or pencil [31, 33], however results of the current study suggest that the type of drawing implement is important to consider for this commonly used test. Use of the stylus vs. fingertip led to improved performance while tracing the Archimedes spiral on the touchscreen tablet for both age groups, and previous studies have also demonstrated that drawing implement impacts performance on other touchscreen tasks [25–29, 43, 44]. For example, middle-aged adults had greater error using the fingertip vs. stylus on a shape tracing task [29] and on gesture strokes [25, 28], while older adults had decreased performance using their fingertip vs. stylus on a drag and drop task [26], and expressed an initial preference for the stylus [43]. The current study extends these findings by implementing the Archimedes spiral tracing task on a popular touchscreen surface to examine age-related changes when tracing with the stylus vs. fingertip, and demonstrates that the type of drawing implement plays a significant role in spiral tracing performance. Young and older adults had decreased performance when tracing with the fingertip vs. stylus. Furthermore, older adults had greater error when tracing with the fingertip compared to young adults, but older adult’s performance was similar to young adults when tracing with the stylus (Fig 2A). These findings suggest the need to carefully consider the type of drawing implement used for the clinical spiral tracing task, as the stylus may not be a sensitive measure of all age-related impairments. Future work may determine whether similar findings exist in patient populations where the Archimedes spiral tracing test is often utilized, for example, in tremor related to Parkinson’s disease [34, 36] and multiple sclerosis [35].

Further analysis was completed to investigate potential mechanisms underlying increased RMS error for older adults while tracing with the fingertip. Given that older adults could perform the tracing task as well as young adults while using a stylus, but that the strength of the relationship between RMS error using the stylus and fingertip was lower for older vs. young adults (Fig 3), other factors are likely influencing older adult’s performance when using their fingertip. We examined the potential influence of five different factors (Table 1). Older vs. young adults demonstrated increased pressing force and reaction time and decreased fingertip sensation, consistent with previous studies [37–39, 45]. However, these age-related differences were not related to increased RMS error for older adults while using the fingertip (Table 1). Older adults reported less touchscreen use and ownership compared to young adults, consistent with a general decline in technology use by older adults [46]. All young adults reported using and owning a touchscreen device while only 46% percent of older adults reported using and 29% reported owning a touchscreen device. Interestingly, RMS error was not different for older adults who had and had not used or owned a touchscreen device. Furthermore, spiral tracing completion time was not different between young and older adults and was not related to RMS error for older adults tracing with the fingertip.

Though the additional variables assessed here did not relate to the age-associated changes in performance, one possible explanation for increased error when tracing with the fingertip is altered visual feedback. A greater segment of the upcoming spiral template may be visible when tracing with the fine stylus tip compared to the larger sized index finger, and the fingertip may occlude a greater area of the display [47–50]. Therefore, the stylus may be better suited for tasks where precise control is necessary [44]. Studies implementing touchscreen icon selection tasks are consistent with this; both increasing target size [49, 51] and offsetting the target from the actual fingertip position [50] to decrease occlusion lead to improved performance. While increased occlusion would have occurred for both age groups when tracing with the fingertip vs. stylus, older adults have been shown to rely on visual feedback more than young adults to modify hand trajectories [52] and decreased visual feedback could have been more detrimental to spiral tracing performance for this age group. Further research is necessary to examine this possibility. For example, if blocking a greater amount of visual feedback during the spiral tracing task by increasing drawing implement size results in greater RMS error scores for older adults, mechanisms related to visual feedback processing may be responsible for the increased RMS error. Altered visual strategies used by older adults have also been shown to impair performance on visuomotor tasks. A greater use of saccadic eye movements in older versus young adults, for example, was related to decreased steadiness on a force-matching task [53], and this altered visual strategy may contribute to increased RMS error during spiral tracing. Future research is necessary to assess age-related differences in visual strategies and their relationship with spiral tracing performance.

Consistent with our expected results, RMS error increased on the low- vs. high-friction touchscreen surface and using the left vs. right hand. It has previously been established that low- vs. high-friction surfaces impair dexterous manipulation, evidenced by decreased performance on a manual dexterity test [17], changes in precision gripping [18, 19], and decreased index finger submaximal force matching [2]. Results of the current study are consistent with this, as RMS error increased on the low- vs. high-friction touchscreen surface for both age groups. RMS error also increased using the left vs. right hand for young and older adults, consistent with studies showing decrements in manual dexterity when using the non-preferred vs. preferred hand [21, 23]. To improve motor performance and facilitate the use of touchscreens, especially for older adults, it may be useful to recommend and/or design interfaces that use: 1) higher friction touchscreen surfaces (i.e. the original iPad glass vs. relatively low-friction screen protectors), 2) a stylus drawing tool, and 3) use of the preferred hand.

The following limitations should be considered. Participants were not constrained to complete the spiral tracing task in a restricted time. Though spiral tracing completion time has been controlled to reduce tremor compensation strategies [30, 35], many studies utilizing Archimedes spiral tracing do not include time constraints [31–34, 36]. We did not impose a time constraint as it could have led to undue stress for some individuals and might deemphasize the goal of the task, to complete the spiral as accurately as possible. Archimedes spiral tracing was utilized based on its ability to detect age-associated changes in hand function, common clinical use, and as a task that both age groups could successfully perform with minimal practice. Continuous tracing required for spiral tracing is one of many gesture types performed on touchscreen surfaces, however, and additional work is needed to examine if these results generalize to other touchscreen gestures. Other constraints may decrease older adults’ use of electronic devices, including decreased self-efficacy and increased anxiety [15, 54]. To take these factors into account, we assessed participant’s previous touchscreen use and ownership, selected a touchscreen task that was easy to understand, and incorporated practice trials for the spiral tracing task. Nonetheless, it is not clear if extensive periods of added practice would have resulted in comparable performance between young and older adults on the tracing task. Despite these limitations, the main results of this study provide important insight into touchscreen design and highlight important factors to consider when using the Archimedes spiral tracing task.
